# DPP-4 inhibitors for treating T2DM - hype or hope? an analysis based on the current literature

**DOI:** 10.3389/fmolb.2023.1130625

**Published:** 2023-05-23

**Authors:** Kunika Saini, Smriti Sharma, Yousuf Khan

**Affiliations:** Molecular Modelling and Drug Design Laboratory, Department of Chemistry, University of Delhi, New Delhi, India

**Keywords:** T2DM, incretin effect, DPP-4 enzyme, DPP-4 inhibitors, (GLP)-1, insulin

## Abstract

DPP-4 inhibition is an interesting line of therapy for treating Type 2 Diabetes Mellitus (T2DM) and is based on promoting the incretin effect. Here, the authors have presented a brief appraisal of DPP-4 inhibitors, their modes of action, and the clinical efficiency of currently available drugs based on DPP-4 inhibitors. The safety profiles as well as future directions including their potential application in improving COVID-19 patient outcomes have also been discussed in detail. This review also highlights the existing queries and evidence gaps in DPP-4 inhibitor research. Authors have concluded that the excitement surrounding DPP-4 inhibitors is justified because in addition to controlling blood glucose level, they are good at managing risk factors associated with diabetes.

## 1 Introduction

The success and ever increasing interest in incretin-based therapies and drugs especially sitagliptin, vildagliptin and exenatide depict their immense importance in improving patient outcomes in T2DM (Nasr and Sadek, 2022). Incretin effect is the enhancement in the secretion of insulin as a function of increase in oral intake of glucose. For a given rise in plasma glucose concentration, the increase in plasma insulin is roughly threefold times greater, if glucose is administered orally compared to intravenous route (Perley and Kipnis, 1967). The main incretin hormones secreted by the epithelial cells of the gastrointestinal track are glucagon-like peptide-1 (GLP)-1 and glucose-dependent insulinotropic peptide, also known as Gastric Inhibitory Polypeptide (GIP), which stimulate the pancreatic cells (α and β cells) and regulate insulin secretion with respect to blood glucose concentration (Drucker, 2006).

The first incretin to be reported was GIP. It is secreted in a single biologically active form by the K-cells in the duodenum and jejunum upon consumption of carbohydrates or lipids. Its main functions are stimulation of insulin secretion in a glucose dependent manner and contribution to fat metabolism in adipocytes. It also has proliferative effect on β-cells. The second important incretin hormone is GLP-1, released from L-cells in the distal ileum and colon (Baggio and Drucker, 2007; Gautier et al., 2008). GLP-1 is similar to but unlike GIP, actions of GLP-1 are better preserved in type 2 diabetes mellitus (T2DM) patients, which makes it a highly desirable target for drug discovery for T2DM (Sharma and Bhatia, 2020); (Nauck et al., 1993).

**FIGURE 1:**
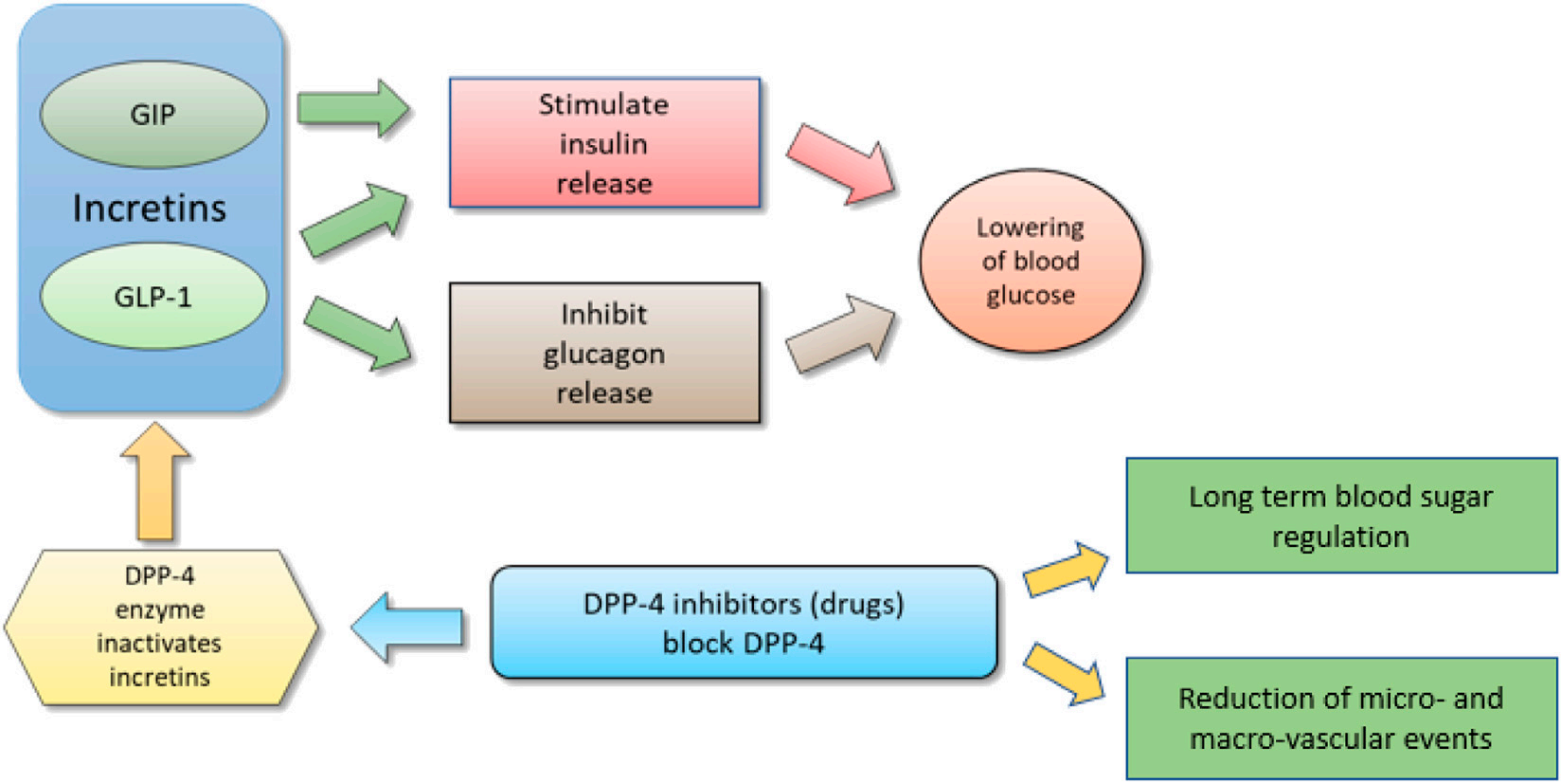
Schematic diagram to show relation between incretins and DPP-4 inhibitors; By I. Karonen under CCA-SA (Karonen, 2007).

However, both GLP-1 and GIP are quickly degraded by dipeptidyl peptidase 4 (DPP-4) enzyme (Figure 1). It cleaves the oligo-peptides after the 2nd amino acid from the N terminal proline or alanine sequences, thus rendering GLP-1 and GIP inactive (Deacon et al., 1995). In addition to that, DPP-4 enzyme also deactivates more than 40 physiologically active hormones, neuropeptides, cytokines, and other proteins *in vivo* through dipeptide cleavage (Mentlein et al., 1993). This very short half-life (<2 min) of GLP-1 due to the action of DPP-4 is a formidable challenge in the incretin-based drug development for T2DM.

DPP-4 is member of a family of proteases which also includes dipeptidyl-peptidase-8 (DPP-8), dipeptidylpeptidase-9 (DPP-9) and fibroblast activation protein (FAP) (Ajami et al., 2004). DPP-4 enzyme is a 766 amino acid trans membrane glycoprotein, also known as adenosine deaminase complexing protein 2 or CD26 (Jose and Inzucchi, 2012). It is a serine amino peptidase enzyme, which exists on the superficial side of epithelial and endothelial cells (involving monocytes and lymphocytes) and propagates in plasma (Fadini and Avogaro, 2011). These enzymes are broadly distributed in various tissues (brain, lungs, kidneys), T-cell, B-cell and natural killer cells (Lambeir et al., 2003). DPP-4 has two mechanisms of action: first as a membrane spanning protein, where it binds and activates adenosine deaminase complexing protein and transfers intracellular signals via dimerization; and second as an enzyme (Lambeir et al., 2003). Thus, a DPP-4 can circulate freely or can exist in the membrane bound form.

### 1.1 Incretin effect in T2DM: GLP-1 receptor agonists vs DPP-4 inhibitors

**FIGURE 2:**
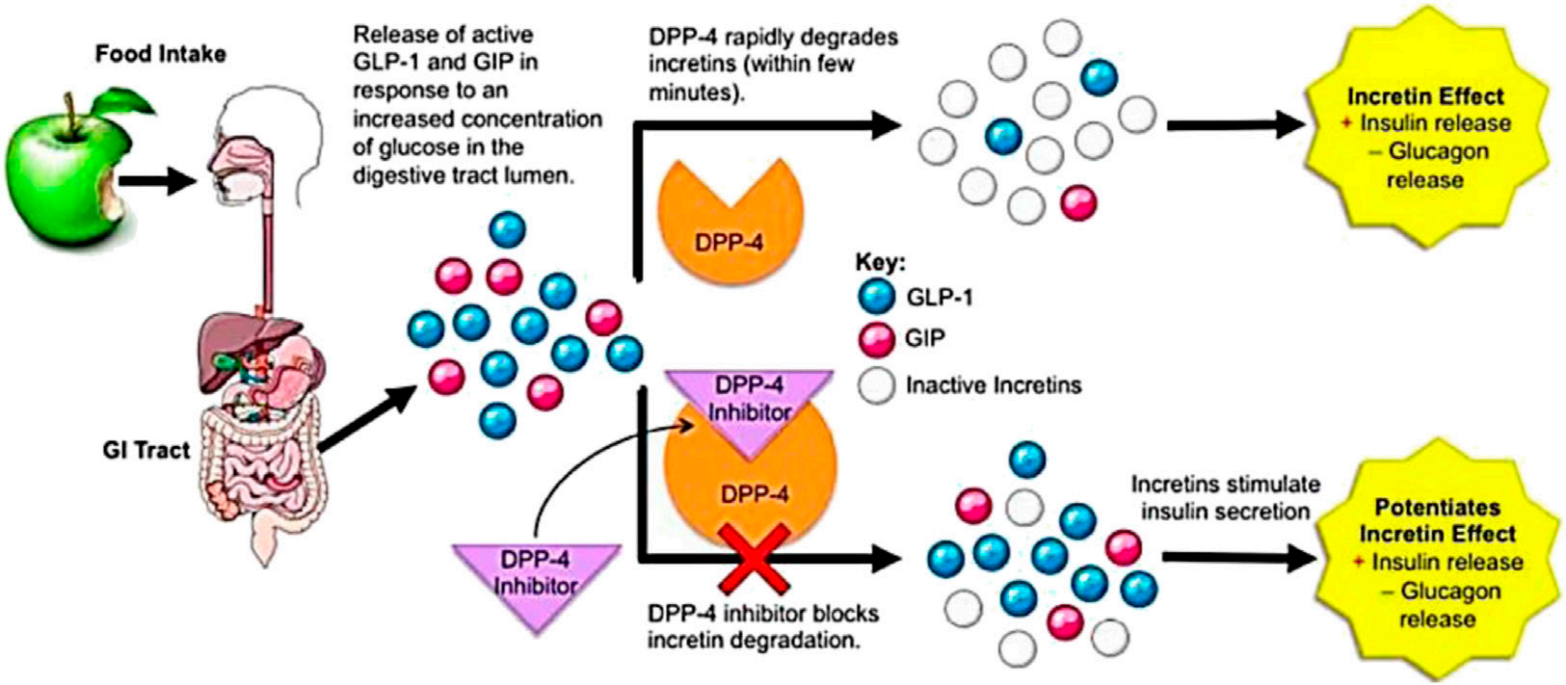
DPP-4 function and mechanism of DPP-4 inhibitors’ action; under CCA-SA; taken from (Makrilakis, 2019).

Broadly, there are two strategies in the drug design for T2DM that make use of the incretin effect. First is the use of GLP-1 receptor agonists (GLP-1RAs) also known as GLP-1 mimetics that enhance GLP-1 action *in vivo* (Parker et al., 2010). These agonists possess sequence similarities to native GLP-1 and can bind and stimulate GLP-1 receptor. Also, they are resistant to DPP-4 action. The second approach is inhibiting the DPP-4 enzyme itself, due to which levels of active GLP-1 increases (Drucker, 2007). The active GLP-1 consists of only one-third to one-half of the postprandial GLP-1 in the plasma and the rest being the inactive fragment (GLP-1 (9–36)amide) formed by truncation. DPP-4 inhibitors raise only the proportion of active GLP-1 rather than total GLP-1 concentration after a meal (Herman et al., 2006a), resulting in elevated plasma levels of GLP-1 at a concentration that does not produce GLP-1-related side effects (Vilsbøll et al., 2001; Sharma and Bhatia, 2021). This is described in Figure 2.

The inhibition of DPP-4 not only elevates the prandial GLP-1 levels but also alters the 24-h pattern of GLP-1 levels, including fasting levels (Mari et al., 2005). This protracted circadian rhythm at a higher level is important for the physiology of incretin receptors (Ahrén, 2007). Hsieh et al. discovered the complimentary action of DPP-4 inhibition and pharmacological enhancement of GLP-1 receptor (GLP-1R) signalling. Multiple studies apart from these, have shown promising positive effects of significant magnitude on blood glucose as well as heart and coronary function (Dicker, 2011). Multiple differences exist between the physiological effects GLP-1 receptor agonists and DPP-4 inhibitors. These are summarised in Table 1.

**TABLE 1:** GLP-1 Receptor Agonists vs. DPP-4 inhibitors.

Parameter	GLP-1 receptor agonists	DPP-4 inhibitors
Total cholesterol	Stimulate effective reduction	Minor reduction only
Weight control	Weight loss	Weight neutral
Safety profile	Gastrointestinal side-effects such as nausea.	Well tolerated
Mode of administration	As injectable peptides (*Except for oral form of semaglutide*)	Small agents can be taken orally

**FIGURE 3:**
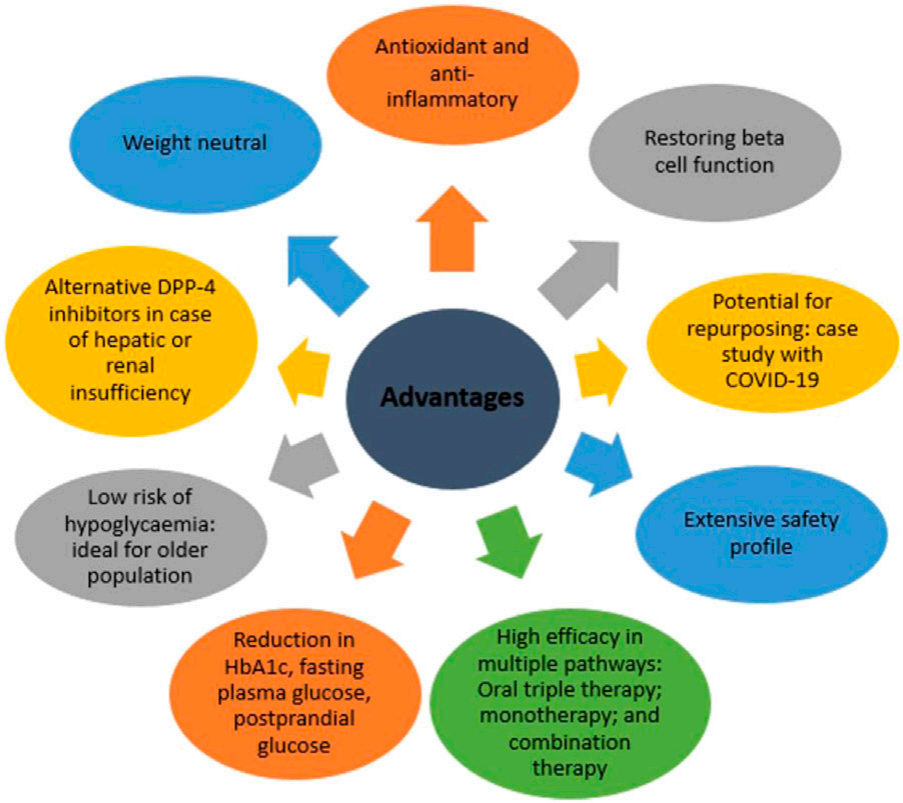
Advantages of DPP-4 inhibitors in clinical practice.

Notably, in a clinical trial which compared short-term 2-week treatment with exenatide (GLP-1RA) *versus* sitagliptin (DPP-4 inhibitor), patient outcomes were better after treatment with exenatide. It was more effective in reducing postprandial glucose, increasing insulin levels, lowering glucagon levels and decreasing caloric intake (DeFronzo et al., 2008a). Despite the apparent better results for GLP-1 RAs, their safety profiles remains a concern. For instance, both exenatide and liraglutide have black box warnings from FDA due to risk of medullary thyroid cancer and pancreatitis in case of exenatide and thyroid C-cell tumours and for patients with multiple endocrine neoplasia syndrome type 2 in case of liraglutide (Pozo et al., 2019). Crucially, DPP-4 inhibitors do not cause nausea or vomiting, which are common side effects of GLP-1 agonists (Ahrén, 2007). Since the DPP-4 inhibitors promote physiological incretin concentrations unlike incretin mimetics which induce pharmacological incretin concentrations. The key advantages of DPP-4 inhibitors are summarized in Figure 3.

One needs to keep in mind that the latest research points that SGLT-2 inhibitors and GLP-1 receptor agonists both reduce cardiovascular events (no study has compared their respective potency in this respect), whereas DPP-4 inhibitors are neutral. However, all over the globe, many research groups are investigating the potential of DPP-4 inhibitors, and so many studies are being published, we felt this topic merited a comprehensive review. Supported by satisfactory performance, obvious gains in the quality of life of a diabetic patient and a robust safety profile, we shall focus on exploring the role of DPP-4 inhibitors as a novel and effective therapeutic strategy for treating T2DM.

## 2 The key interactions between the ligand and DPP-4 complex

**FIGURE 4:**
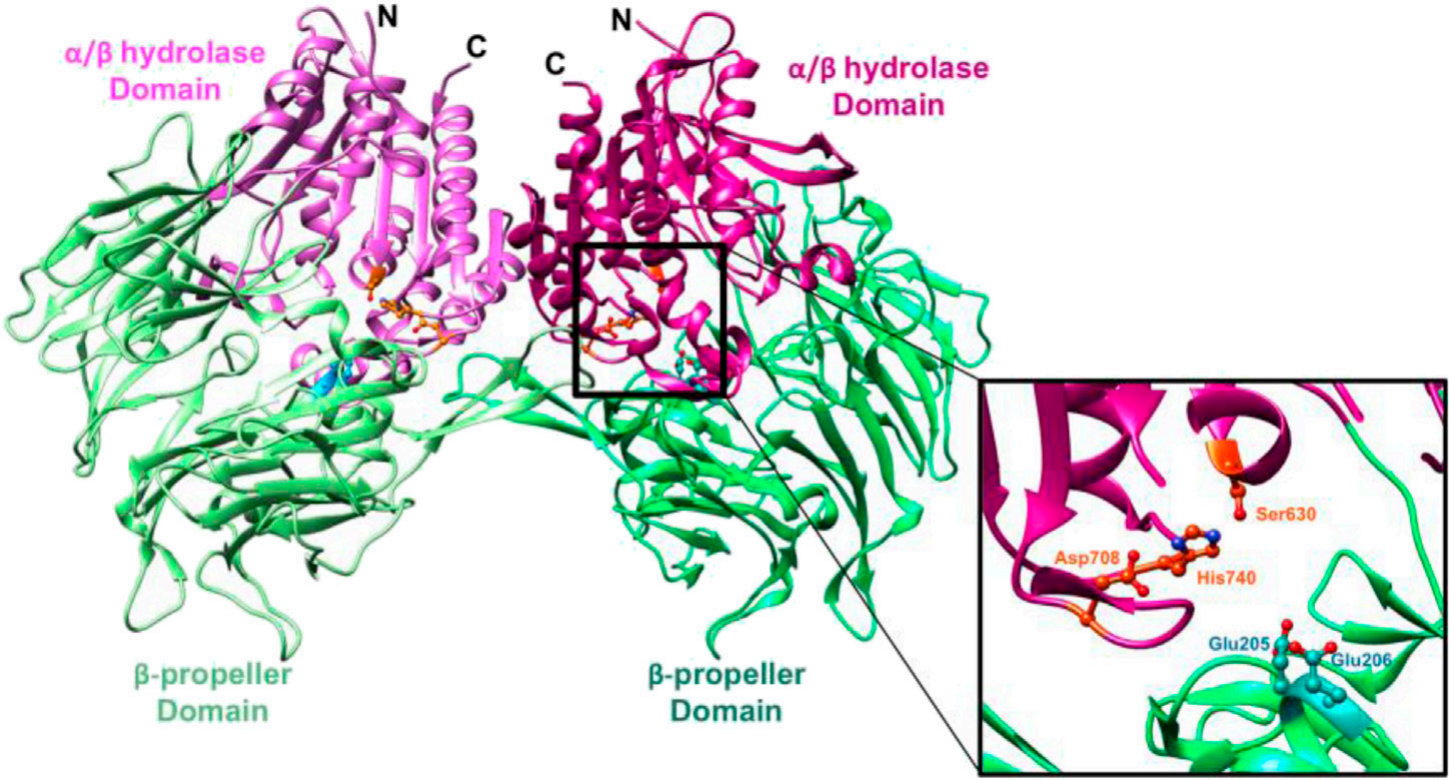
(Metzler et al., 2008): Ribbon representation of the structure of the symmetric DPP-4 homodimer. “α/β hydrolase domain and the β-propeller domain of each monomer unit are coloured in two shades of pink and green respectively (PDB ID 1r9m). The inset shows a closeup of the DPP-4 active site (corresponding to the right half) of the DPP-4 homodimer. The catalytic triad (Ser630, His740 and Asp708) is shown in orange and the anchoring Glu motif (Glu205/Glu206) in cyan”.

As shown in Figure 4, the β-propeller domain is packing against the α/β hydrolase domain. In addition to that, at the interface of β-propeller domain and α/β hydrolase domain, catalytic triad (residues Ser^630^, His^740^ and Asp^708^) is located (Aertgeerts et al., 2004), with both these domains participating in the binding of the inhibitor. In the DPP4-like protein family, glutamic acid-rich loop (Glu^205^/Glu^206^) is highly conserved and is responsible for substrate recognition. The catalytic serine S^630^ falls within the pentapeptide sequence Gly^628^-X-Ser^630^-Tyr^631^-Gly^632^, which is highly conserved across the α/β hydrolase protein family and is indispensable for catalytic activity.

**FIGURE 5:**
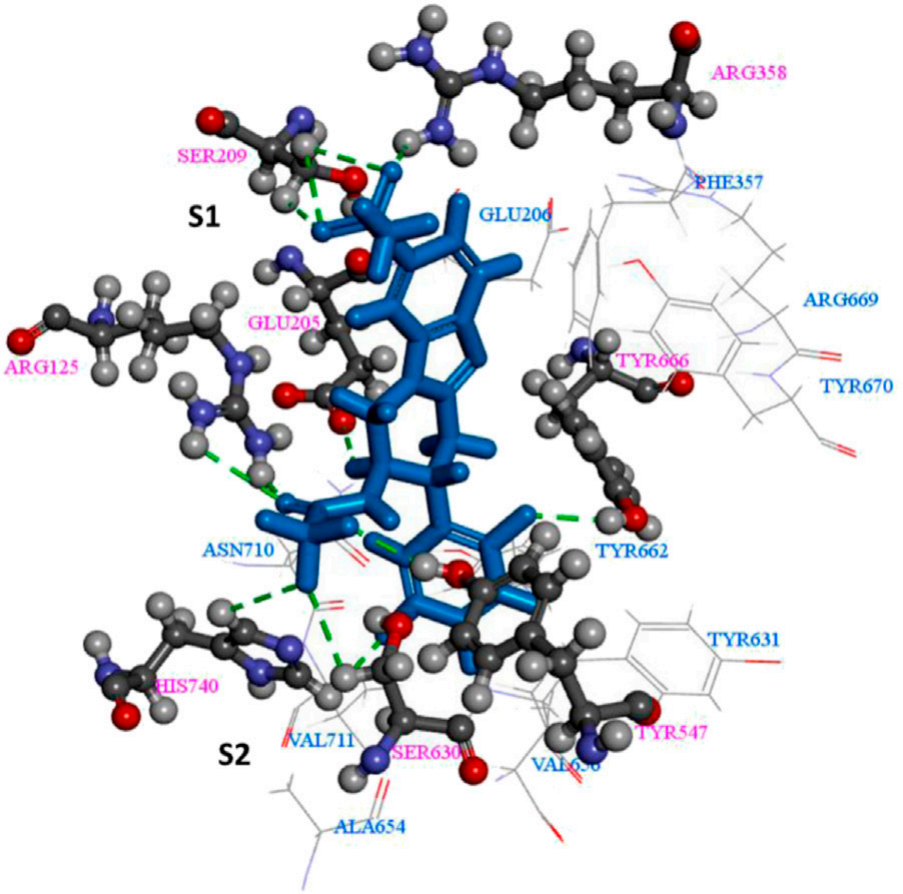
The key interactions between the ligand and DPP-4 complex (Meduru et al., 2016).

Studying the binding interactions of DPP-4 inhibitors at the binding site are crucial in understanding their performance and also for guiding research into novel drug candidates. The DPP-4 binding site can be divided into two pockets *viz.* S1 and S2 and a hydrophobic sub-S1 or sub-S3 sub-pocket. Figure 5 represents a general DPP-4 ligand. It contains an aromatic group, a heterocycle, a primary amine, as well as a nitrile group. S1 pocket is usually occupied by the aromatic ring or the saturated heterocycle, whereas the primary amine group forms hydrogen bond with Glu^205/206^ and Tyr^662^. S2 site binds with the aromatic heterocycle or fused rings present in the inhibitor (Metzler et al., 2008). The hydrophobic nature of the sub-pockets dictate that these are occupied by an aromatic group, and linker is necessary between the S1 and sub-S1 binding ligands (Deng et al., 2018). A common feature amongst most DPP-4 inhibitors approved by regulatory authorities is the presence of a cyanopyrrolidine moiety. The presence of the nitrile group allows the ligand to catalytically bind to the active serine hydroxyl group (Kumar et al., 2021).

Their main interaction is with the DPP-4 complex which are recognized for competitive inhibition (Jose and Inzucchi, 2012). DPP-4 enzyme inhibition also regulates the action of various cardio active elements, neuropeptide Y and stromal cell derived factor-1 (SDF-1) (Drucker, 2007). Fibroblast activation protein, DPP-2, DPP-8 and DPP-9 show DPP-4 enzyme like activity and therefore DPP-4 inhibitors must be selective (Green et al., 2006).

## 3 DPP-4 inhibitors: current status

DPP-4 inhibitors are already being employed in T2DM treatment globally. Table 2 lists all the DPP-4 inhibitors till date in various stages of drug development (Kumar et al., 2021).

**TABLE 2:** List of DPP-4 inhibitors (Kumar et al., 2021).

S.No.	DPP-4 inhibitors	Name		Year of approval
1	Approved Augeri et al. (2005), Hulin et al. (2005), Del Prato (2007), Thornberry and Weber (2007), Yoshida et al. (2012), Burness (2015), Nishio et al. (2015), Grimshaw et al. (2016), Kim et al. (2016), Tan and Hu (2016), Shiheido-Watanabe et al. (2021)	i.	Sitagliptin phosphate monohydrate	2006 (United States)
		ii.	Vildagliptin	2007 (European Union)
		iii.	Saxagliptin hydrochloride	2009 (United States)
		iv.	Alogliptin Benzoate	2010 (Japan)
		v.	Linagliptin	2011 (United States)
		vi.	Gemigliptin L-tartrate sesquihydrate	2012 (South Korea)
		vii.	Anagliptin	2012 (Japan)
		viii.	Tenegliptin Hydrobromide Hydrate	2012 (Japan)
		ix.	Denagliptin tosilate	2014 (United States)
		x.	Omarigliptin	2015 (Japan)
		xi.	Evogliptin Hydrochloride	2015 (South Korea)
		xii.	Trelagliptin Succinate	2015 (Japan)
2	In phase 3 clinical trials	i.	Fotagliptin Benzoate	NA
		ii.	CPL 2009–0031	NA
		iii.	DBPR-108	NA
3	In phase 1 clinical trials	i.	DC291407	NA
		ii.	Augliptin Hydrochloride	NA
		iii.	Yogliptin	NA
		iv.	HD118	NA
		v.	ARI-2243	NA
		vi.	Cetagliptin phosphate	NA
		vii.	PBL-1427	NA
		viii.	HSK-7653	NA
4	In phase 2 clinical trials	i.	Besigliptin Tosilate	NA
		ii.	Imigliptin Hydrochloride	NA
5	NDA filed	i.	Gosogliptin Hydrochloride	NA
		ii.	Retagliptin Phosphate	NA

DPP-4 inhibitors are characterized into three different classes based on their binding sites (refer to Figure 5 from Section 2) (Tomovic et al., 2018). Vildagliptin and saxagliptin make up Class 1 where binding occurs only on the S1 and S2 binding sites between the nitrile group of the cyanopyrrolidine group and Ser^630^ of the active site on DPP-4. Saxagliptin is five times more active in blocking DPP4 than vildagliptin. Class 2 is composed of linagliptin and alogliptin where binding interaction with the S1 sub pocket or in case of the former with S2 sub pocket as well, giving it eightfold higher activity than alogliptin. The uracil rings in both ligands force a conformational alteration of Tyr^547^ within the S2 sub pocket. Class 3 consists of ligands binding with the S2-extensive site. Sitagliptin and tenegliptin form this class which has the highest inhibitory capability among gliptins (Röhrborn et al., 2015).

Sitagliptin, alogliptin and linagliptin possess high selectivity for DPP-4 due to strong binding with S2-extensive site which is absent in related peptidases like DPP-8, DPP-9, and FAP, wherein vildagliptin seems less selective. These inhibitors are appropriate for once or twice daily dosage, orally active are absorbed relatively fast. They are all excreted renally after negligible metabolism, except linagliptin which is eliminated via the biliary pathway (Deacon, 2019; Gallwitz, 2019). Insulin secretion can be enhanced by sulphonylureas independently and therefore introduce diabetic patients to a heightened risk of hypoglycaemia. DPP-4 inhibitors are thus added to the sub-maximal limit of sulphonylurea to achieve tighter glycaemic control (Gerich, 2010). In fact, DPP-4 inhibitors are now replacing the use of sulphonylureas as insulin-releasing agents due to their insulin-tropic effect, and notably because DPP-4 inhibitors reduce the intrinsic risk of low blood sugar or hypoglycaemia. Also, DPP-4 inhibitors are body weight neutral, in contrast to sulphonylurea therapy which is linked with body weight gain (Palmer et al., 2016). Differences between DPP-4 inhibitors with various classes of antidiabetic agents are shown in Table 3. Prior experiments *in vitro* have exhibited that DPP-4 inhibitors impede T-cell proliferation and cytokine expression and these agents have also been examined in animal models, showing potential anti-inflammatory effects (Yazbeck et al., 2009).

**TABLE 3:** Comparison of DPP-4 inhibitor with various classes of antidiabetic agents.

Class of anti-diabetic agents	Action mechanism	Change in body weight	HbA1c reduction (%)	Risk of hypoglycaemia	Microvascular and macrovascular events
DPP-4 Inhibitors	Inhibits DPP-4 enzyme action; elevates postprandial incretin concentration	Weight neutral (−0.09 to +1.11 kg) Lozano-Ortega et al. (2016)	0.5–0.8	Low	Neutral for cardiovascular events; might cause congestive heart failure by degradation of B-type natriuretic peptide
Insulin	Activates receptors of insulin and triggers downstream signaling in various sensitive tissues	Weight gain (+1.56 to +5.75 kg) Giugliano et al. (2011)	1–2.5	Evident	Might lead to heart failure when associated with thiazolidinedione
Metformin	Insulin sensitizer; various impacts on suppression of hepatic glucose production	Change in body weight (+1.5 to −2.9 kg) Golay (2008)	1–2	No	Lowers coronary deaths by 50% and myocardial infarction by 39%
Thiazolidinedione	True insulin sensitizer; activate nuclear transcription factor PPAR-γ	Weight gain (+2.30 to +4.25 kg) Lozano-Ortega et al. (2016)	0.5–1.4	Low	Pedal edema, cardiac failure
Sulphonylureas	Elevates insulin secretion; closes K_ATP_ channels on plasma membrane of β-cell	Weight gain (+1.99 to +2.31 kg) Hirst et al. (2013)	1–2	Evident	Elevated cardiovascular risk factors, majorly because of hypoglycaemia
GLP-1 Analogues	Activates receptors of GLP-1; elevation in insulin secretion, suppression in glucagon secretion, and gastric emptying is delayed	Reduction in body weight (−1.14 to −6.9 kg) Trujillo et al. (2015)	0.5–1.5	No	Lowers cardiovascular risk
SGLT-2 Inhibitors	Insulin-independent mechanism of action; glucosuria because of inhibition of reabsorption of glucose in renal proximal tube	Reduction in body weight (−0.9 to −2.5 kg) Storgaard et al. (2016)	0.5–0.9	Low	Positive cardiovascular impact because of lowering of uric acid and sodium absorption, and decrease of blood pressure

Sitagliptin was the first commercialized, orally active and selective DPP-4 inhibitor (Dhillon, 2010), which was capable of stimulating a 2-fold increase in post-meal active plasma GLP-1 levels with respect to placebo (Lim et al., 2009). It is presently available as a single agent or in fixed-dose association with metformin (Reynolds et al., 2008). A 50 mg daily dosage produces 80% inhibition of DPP-4 activity in healthy subjects and type 2 diabetic patients (Herman et al., 2006a). It is extremely selective for DPP-4 (upto 2600 times higher than other DPP family enzymes (Richter et al., 2008a). Additionally, sitagliptin improves surrogate markers of β-cell function in humans and enhanced β-cell mass in animal studies (Palalau et al., 2009).

Saxagliptin is an incredibly selective and reversible DPP-4 inhibitor (Tahrani et al., 2009a). It can increase the GLP-1 levels to 1.5 to 3-fold with a single dose of 2.5–400 mg which inhibits DPP-4 activity between 50% and 79% (Chacra, 2010). Saxagliptin is tenfold more effective than vildagliptin and sitagliptin. Its pharmacokinetics permit once-daily administration and are not influenced by age or gender. The pharmacokinetics of other drugs are also not affected by combination of saxagliptin with some other (oral antidiabetic agents) OADs such as metformin, pioglitazone, or glyburide in a remarkable way (Tahrani et al., 2009a). Saxagliptin improved β-cell function when employed as mono-therapy or in combination with metformin (Jadzinsky et al., 2009). However the similar improvement was not seen when combined with suboptimal glyburide (Chacra et al., 2009). The FDA endorsed saxagliptin for its use as a supplement to diet and exercise to improve glycaemic control in adults for curing type 2 diabetes (Anz et al., 2014). Saxagliptin can be employed in mono-therapy or in combination with other OADs including metformin, sulphonylureas or TZDs (Karyekar et al., 2011).

Vildagliptin is another selective DPP-4 inhibitor that can produce 1.5- to 3-fold increase in post meal active plasma GLP-1 levels *vis-à-vis* placebo (Hu et al., 2009). A 100-mg vildagliptin dose is enough to completely suppress the activity of DPP-4 in patients with T2DM (Balas et al., 2007). Vildagliptin also improved β-cell replacement function in humans and stimulated β-cell mass increase in animal studies (Mari et al., 2008). It improved HbA1c and reduced FPG and PPG in type 2 diabetic patients when used in mono-therapy or in combination with some other OADs. With vildagliptin the improvement of glycaemic control lasts at least 2 years (Scherbaum et al., 2008), and is better in patients with greater initial HbA1c levels (Rosenstock et al., 2007). Vildagliptin has also been identified to be effective in the older population (Das et al., 2021). Due to their compatible mechanisms of action and low risk of hypoglycaemia, vildagliptin and metformin have been formulated in a single tablet (Tahrani et al., 2009b).

Alogliptin is another effective, highly selective DPP-4 inhibitor approved in 2010. It can be employed in mono-therapy or in combination with metformin or glyburide for T2DM patients. The improvements in fasting glycaemia and HbA1c are maintained for at least 26 weeks. Alogliptin possesses good gastrointestinal tolerability, is weight neutral and has a very low degree of hypoglycaemia risk (Nauck et al., 2009). Administration of alogliptin with metformin, pioglitazone or glibenclamide in healthy patients suggests no significant drug-drug interactions (Karim et al., 2009). Alogliptin in combination with a specific dose of insulin, improved glycaemic control without elevating hypoglycaemia rates and without magnifying weight gain (Rosenstock et al., 2009a). Notably, greater reductions in HbA1c levels were identified when alogliptin was combined with pioglitazone, without elevating the possibility of hypoglycaemic events (Deacon, 2011). The therapeutic effectiveness of alogliptin-pioglitazone combination deserves attention in the future management of T2DM (Argyrakopoulou and Doupis, 2009).

Other important approved DPP-4 inhibitors as listed in Table 2 are Linagliptin, Gemigliptin L-tartrate sesquihydrate, Anagliptin, Tenegliptin Hydrobromide Hydrate, Denagliptin tosilate, Omarigliptin, Evogliptin Hydrochloride and Trelagliptin Succinate.

## 4 Pharmacological variation among different DPP-4 inhibitors

**FIGURE 6:**
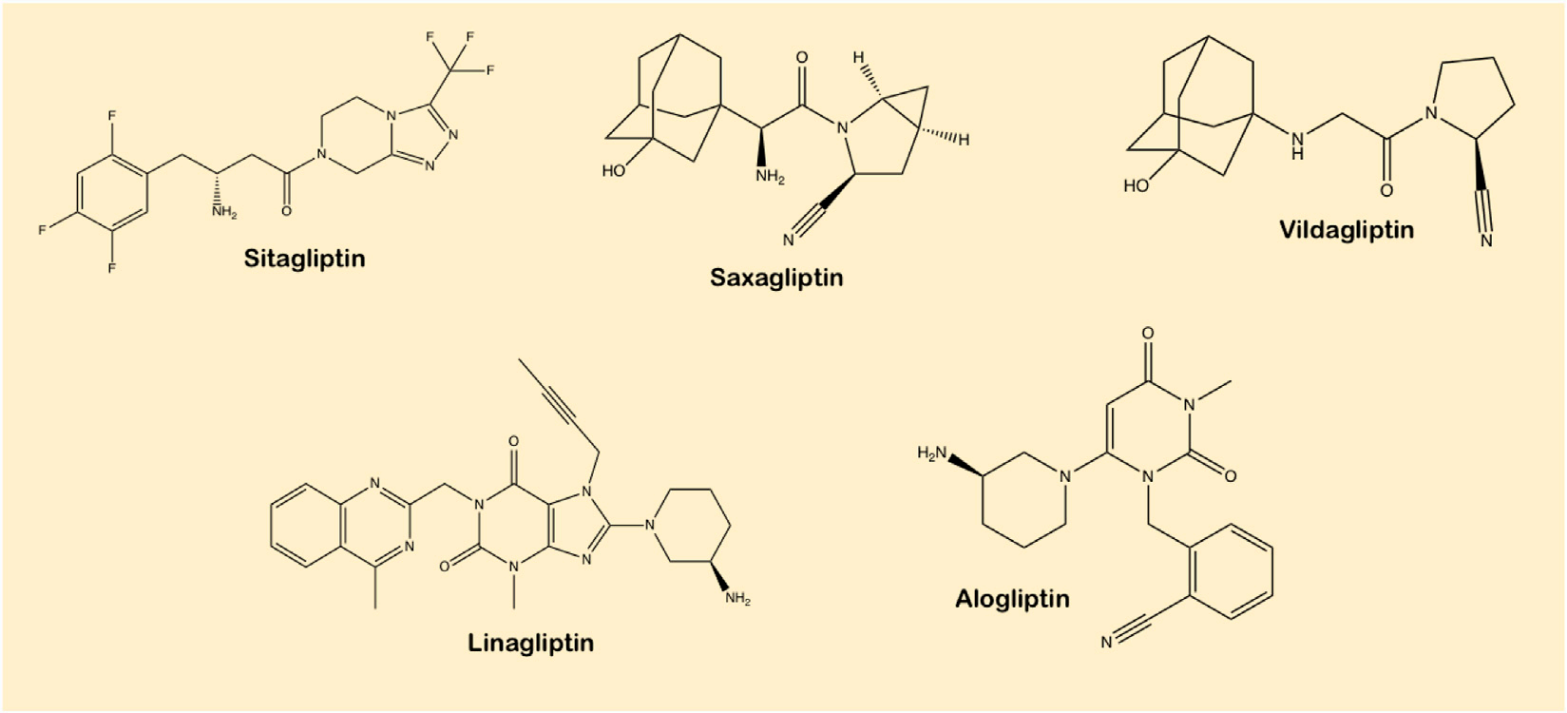
Structural diversity in DPP-4 inhibitors.

As can be seen from Figure 6, DPP-4 inhibitors constitute a heterogeneous class of small molecules with diversity and variations in chemistry, in pharmacokinetic properties such as absorption, distribution, metabolism and excretion routes, and in pharmacodynamic components such as potency and selectivity of DPP-4 inhibition (Scheen, 2012a). From a pharmacological point of view, tight-binding inhibitors are crucial as once they are bound to their target, inhibition of the enzyme operation continues even after the loose drug has been removed from circulation or from the vicinity of the site of action, a profile which is ideal for once-daily dosing. Currently available DPP-4 inhibitors display slow, tight-binding inhibition kinetics and are reversible competitive inhibitors of DPP-4 (Villhauer et al., 2003).

Despite clinical dissimilarities within the group, all DPP-4 inhibitors have a common working action. Firstly, all DPP-4 inhibitors possess pronounced structural heterogeneity which may account for differences in the pharmacokinetic properties. Linagliptin, for instance, possesses distinctive xanthine-based structure (Deacon and Holst, 2010). Its terminal half-life of 184 h (Hüttner et al., 2008), is substantially longer than that of sitagliptin (10–12 h) (Neumiller, 2009). Saxagliptin assimilation by CYP3A4/5 produces an active metabolite, however metabolites of linagliptin, vildagliptin and sitagliptin seem to be inactive. Also, terminal half-life of saxagliptin is 2.5 h, notably less than the mean half-life of 26.9 h for plasma DPP-4 inhibitors. A direct consequence is that if patients miss a dose, the long terminal half-life of linagliptin would predictably retain effectual glycaemic control whereas the short half-lives of sitagliptin and saxagliptin indicate that patients need to strictly follow the dosing interval (Gerich, 2010). When handling T2DM patients with renal dysfunction, the differences in the path of metabolism of action may also prove pivotal. Linagliptin is unlikely to accrue in renally impaired T2DM patients unlike other DPP-4 inhibitors that are removed renally. Due to the strong binding capacity of linagliptin with DPP-4, it rapidly saturates at low doses (Fuchs et al., 2009) and therefore any free linagliptin not bound to DPP-4 is quickly eliminated (Ahmad, 2007). Linagliptin is primarily excreted unchanged by enterohepatic mechanism and thus is appropriate in renally impaired patients. Vildagliptin, however, is excreted *via* the kidney by instant breakdown into an active metabolite, with an absolute bioavailability being 85% (He, 2012).

### 4.1 Pharmacokinetic profile

DPP-4 inhibitors and other oral hypoglycaemics such as metformin, sulphonylureas or thiazolidinediones have not exhibited any concerted pharmacokinetics (Graefe-Mody et al., 2011). There are no prominent interactions with lipid reducing agents (Scheen, 2012b) or with hormonal contraception (Friedrich et al., 2011). Anticoagulation potency of warfarin is not affected (Wright et al., 2009). Dose adjustment of digoxin is not recommended for administration of DPP-4 inhibitors (He et al., 2007a). The pharmacokinetic properties of DPP-4 inhibitors qualify for a once-daily dosing regimen (Herman et al., 2005), although greater glycaemic effect is present if given twice a day. In type 2 diabetes patients and healthy volunteers, studies have shown that these inhibitors are rapidly absorbed (c_max_ is 1–2 h) with explicit oral bioavailability of 80%–85% (Herman et al., 2005). Modest hepatic insufficiency has no clinical effect on the pharmacokinetic properties of sitagliptin (Zerilli and Pyon, 2007). The pharmacokinetic properties of vildagliptin and sitagliptin are independent of BMI. And the IC_50_ value for sitagliptin, vildagliptin and saxagliptin for inhibiting DPP-4 enzyme is displayed in Table 4.

**TABLE 4:** IC_50_ value for inhibiting DPP-4 enzyme.

DPP-4 inhibitors	IC_50_ (nm) for DPP-4 enzyme
Sitagliptin Davis et al. (2010)	18
Vildagliptin Brandt et al. (2005)	3.5
Saxagliptin Kim et al. (2006)	26

No clinically apparent pharmacokinetic effects were displayed by linagliptin *vis-à-vis* commonly prescribed oral hypoglycaemics such as metformin, pioglitazone, glibenclamide or with medications commonly employed for patients with cardiac disorders such as warfarin, digoxin (Neumiller, 2012). The differences in the binding and metabolic characteristics of DPP-4 inhibitors partly explains the dosing frequency of all of them, except vildagliptin. Saxagliptin is advised because of the existence of its active metabolite, which continues to have half the potential of saxagliptin. Whereas, vildagliptin requires twice daily administration. These drugs possess reversible and low binding to plasma proteins, with the exception of linagliptin (70%) and alogliptin (∼100%) and the latter are poorly metabolized (Scheen, 2012a). For saxagliptin, the metabolism is mediated in liver by CYP3A4 and CYP3A5 isoenzymes. Thus, strong inhibitors and prompters of these isoenzymes can affect the pharmacokinetic features of saxagliptin (Scheen, 2010a). Vildagliptin undergoes liver metabolism and produces multiple inactive metabolites but is not mediated by CYP (Deacon, 2011). And linagliptin is eliminated mostly in the faeces, a dose adjustment is not required according to the stage of creatinine clearance (Ceriello et al., 2014). Pharmacokinetic outline of DPP-4 inhibitors depicts negligible risk of drug interactions (Scheen, 2010b), which is significantly advantageous in patients undergoing treatment with complex drug systems (Deacon, 2011). Also metformin did not alter the pharmacokinetics of sitagliptin (Herman et al., 2006b). It is possible that potential cytochrome P450 3A4 (CYP3A4) inhibitors such as ketoconazole, itraconazole, ritonavir and clarithromycin could affect the pharmacokinetics of sitagliptin in severely renal insufficient patients (Fasanya, 2021). In case of saxagliptin, significant pharmacodynamics interactions were not observed between saxagliptin and its metabolite. However, when co-administered with ketoconazole and diltiazem, interactions were observed and so dose adjustment is required (Tahrani et al., 2009a). When vildagliptin was used with other OADs such as digoxin, warfarin, simvastatin, amlodipine, ramipril or valsartan no clinically pertinent pharmacokinetics interactions were observed (Ayalasomayajula et al., 2007). Since vildagliptin is employed in combination with metformin in patients with T2DM, either independently or in a fixed-dose association, it is necessary to analyse the pharmacokinetic inter-effects of these two compounds, as they may significantly influence glycaemic control (Tahrani et al., 2009b). Also, the combination of DPP-4 inhibitors with pioglitazone was well tolerated. Therefore, vildagliptin may be advised in association with metformin, thiazolidinedione or sulphonylurea for the management of T2DM patients when monotherapy is insufficient and not satisfactory (Banerjee et al., 2009).

### 4.2 Pharmacodynamic profile

In accordance with their chemical structure, DPP-4 inhibitors are categorized into peptidomimetics like sitagliptin, saxagliptin and vildagliptin and non-peptidomimetics including linagliptin and alogliptin (Scheen, 2010b). Sitagliptin possess a triazolopiperazine-based structure; vildagliptin and saxagliptin have cyanopyrrolidine-based structure, whereas alogliptin and linagliptin have pyrimidinedione-based and imidazole-based scaffolds, respectively (Havale and Pal, 2009). Linagliptin is most effective in the inhibition of enzyme as compared to other DPP-4 inhibitors. The prolonged action is directly related to the strength and reversibility of binding to the enzyme (Ligueros-Saylan et al., 2010). DPP-4 inhibitors bind at a similar site as the fragment of biochemically relevant substrate (Berger et al., 2018). Sitagliptin is classified as a ‘competitive enzyme inhibitor’ that inhibits the enzyme in a dose-dependent mode and its dissociation is instant, whereas vildagliptin and saxagliptin inhibit the DPP-4 enzyme in a biphasic process, where the initial formation of the reversible covalent enzyme-inhibitor complex is pursued by a slow dissociation. In this case, they act as a ‘substrate-blocker’. As a repercussion, the enzyme inhibition is sustained even after elimination of the free drug and explains the longer duration of action of vildagliptin and saxagliptin in spite of their short half–life (Deacon, 2011).

### 4.3 Other pharmacological differences

Differences among all DPP-4 inhibitors have been observed in the effects on DPP-4 inhibition, GLP-1, insulin and glucagon levels and in the antioxidant activity.

#### 4.3.1 DPP-4 inhibition

The administration of single-dose DPP-4 inhibitors provides long-lasting effects in both healthy volunteers and type 2 diabetes patients (Ahrén et al., 2004). The inhibition of DPP-4 increases with dose. The extent and duration of inhibition is not altered by age, gender or body mass index.

#### 4.3.2 Antioxidant activity

Matsui et al. highlighted that vildagliptin downregulates generation of oxidative stress and reduces vascular injury in thoracic aorta of fatty rats, but there is no explanation for the glucose-reducing-independent effects of vildagliptin on vascular injury (Matsui et al., 2011). Linagliptin is also known to propagate pleiotropic vasodilatory, antioxidant and anti-inflammatory effects in animal models. Similar results have also been observed in a randomised trial in humans, where linagliptin improved vascular function and decreased inflammation markers (Baltzis et al., 2016).

#### 4.3.3 GLP-1

One of the important roles of DPP-4 is to inactivate GLP-1. GLP-1 lowers post prandial glucose (PPG) by stimulating secretion of insulin, inhibiting release of glucagon and delaying gastric emptying (Nicolaus et al., 2011). Vildagliptin and sitagliptin studies have reported increased levels of active GLP-1 both at baseline and in reaction to a meal (during postprandial period) which suggests that the effect of DPP-4 inhibitors on glucose tolerance is interspersed by increased levels of active GLP-1 (Herman et al., 2006a).

#### 4.3.4 Insulin and glucagon

Improved metabolic control by DPP-4 inhibition in T2DM patients is linked with reduced glucagon levels and, in spite of the lower glycaemia, unaltered insulin levels (Ahren et al., 2004). A greater suppression of glucagon was observed with vildagliptin than with sitagliptin in the course of inter-prandial periods, however these differences were not noteworthy in postprandial periods (Marfella et al., 2010).

#### 4.3.5 β-cell function

Many investigators have concluded that DPP-4 inhibitors can improve β-cell function in humans as evidenced by increased insulin secretion with DPP-4 inhibitors. It is reported that vildagliptin and sitagliptin significantly increased the insulin secretion rate as compared to placebo (Barnett, 2006). This is because both these agents are very effective in preventing the degradation of endogenous GIP and GLP-1. Thereby improving the levels of active incretin, which further stimulates pancreatic insulin secretion and inhibits glucagon release.

#### 4.3.6 Microvascular and macrovascular events

Microvascular events are nephropathy, retinopathy, diabetic ulcers, and peripheral and autonomic neuropathy. Diabetes might cause serious issues like cardiovascular effects (cardiovascular mortality, stroke, myocardial infarction), renal disorder and lower-extremity detachment. FDA completed cardiovascular outcomes trials for all four US-approved DPP-4 inhibitors and it was found that all DPP-4 inhibitors possess a neutral impact on microvascular and macrovascular events except saxagliptin, which might elevate the heart failure risk. Also, few research have displayed that linagliptin and saxagliptin might slow down the albuminuria progression in T2DM patients (Taylor and Lam, 2020).

#### 4.3.7 Sarcopenia and other muscle disorders

Sarcopenia is described by a reduction in muscle strength, mass, and quality. It is commonly attributed to ageing and various chronic disorders like T2DM. A study shows that insulin is capable of lowering sarcopenia risk, whereas DPP-4 inhibitors show a neutral effect (Massimino et al., 2021). However, in another study, DPP-4 inhibitors show a positive impact on the reduction of various sarcopenic parameters like gait speed, skeletal muscle strength, muscle mass, and associated indices (Rizzo et al., 2016).

## 5 Safety profile of DPP-4 inhibitors

DPP-4 inhibitors exhibit appreciable safety and tolerance profile. Some recurrent adverse effects are reported such as nasopharyngitis and skin lesions but these are not serious enough to lead to discontinuation of the treatment.

**FIGURE 7:**
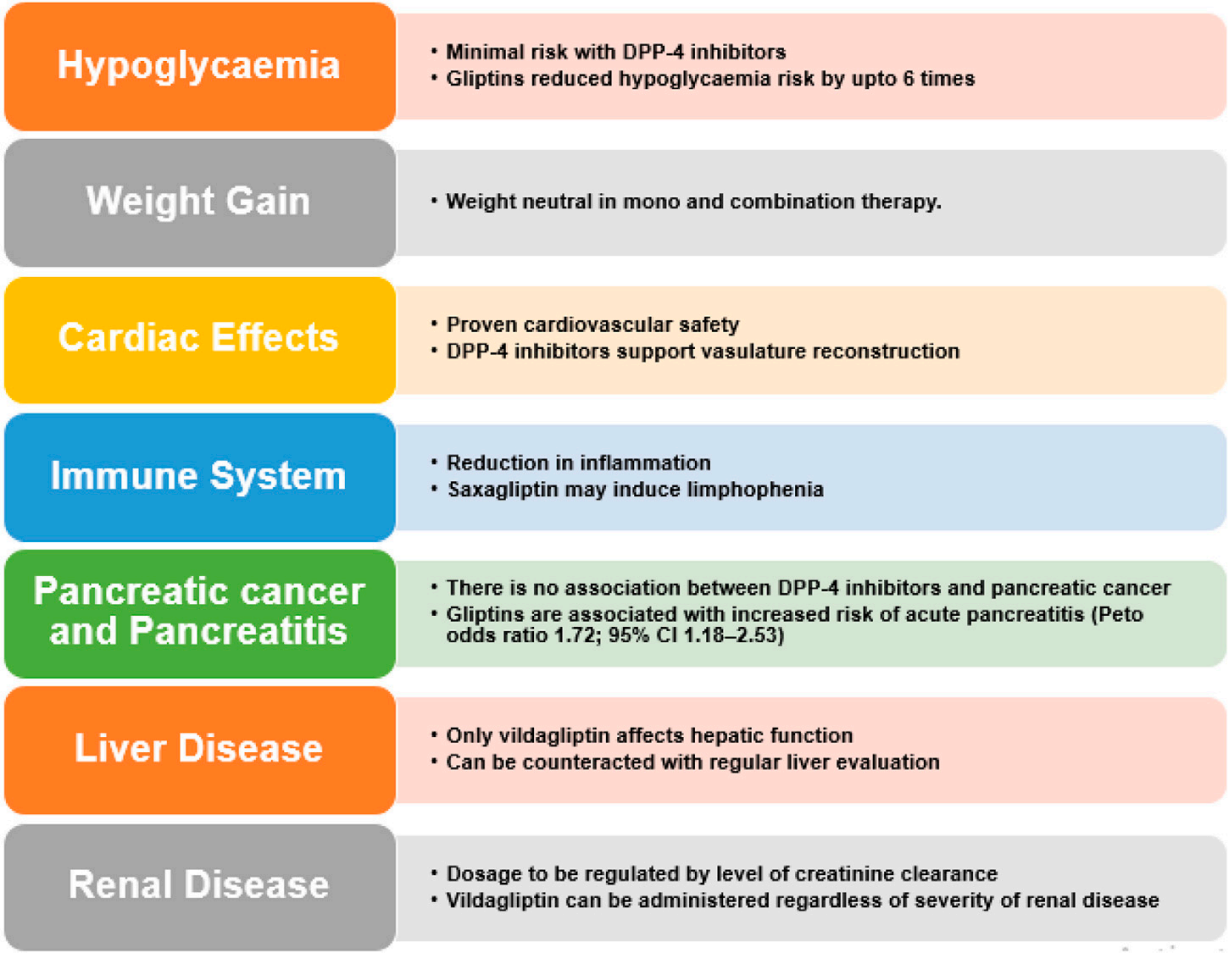
Safety profile of DPP-4 inhibitors.

The effectiveness and safety profile of these inhibitors manifests a favourable scenario particularly for elderly subjects, but only when any influence on complications is not needed (Gallwitz, 2016; Sesti et al., 2019). Other side effects include infection in upper respiratory tract, urinary tract infection and headache. Urticarial dermatological reactions and angioedema have also been stated. Also, T2DM patients face twice the risk of acute pancreatitis and various retroactive studies and meta-analysis indicate that DPP-4 inhibitor therapy actually lowers said risk (Pinto et al., 2018). These safety profile of DPP-4 inhibitors is summarized in Figure 7.

DPP-4 has consequences beyond its proteolytic action which includes T-cell activation and proliferation (Lambeir et al., 2003). DPP-4 inhibitors may prolong the action of various hormones. The potential side-effects associated with the action of these hormones include neurogenic inflammation, elevated blood pressure and magnified general inflammation and allergic reactions (chemokines). Such side-effects have not been observed in preclinical or clinical research yet.

Linagliptin (Forst et al., 2010), saxagliptin (Rosenstock et al., 2009b), vildagliptin (Pi-Sunyer et al., 2007), sitagliptin (Aschner et al., 2006), and alogliptin (DeFronzo et al., 2008b) shows mild tolerability with adverse event profiles compared to placebo. Currently, linagliptin is the only DPP-4 inhibitor that showed improvement in wound healing in preclinical studies (Gupta and Kalra, 2011). The wide distribution of DPP-4 in various tissues has led to concern that they may have increased risk of infectious and inflammatory processes. DPP-4 adjusts levels of various mediators required in immune modulation and a meta-analysis report identifies elevated risk of infections with sitagliptin, but not with vildagliptin (Richter et al., 2009). A systematic review disclosed that DPP-4 inhibitors slightly increase the risk of nasopharyngitis and urinary tract infections in contrast with non–incretin-based placebo or OAD (Amori et al., 2007). Nasopharyngitis is detected in ≥5% of sitagliptin cured patients (Fasanya, 2021).

### 5.1 Hypoglycaemia

As the glucose reducing effects of GLP-1 are dependent on elevated blood glucose that fall back to normal, the hypoglycaemia risk during DPP-4 inhibitor dosing is minimal. They follow a glucose-dependent mechanism, i.e., they enhance insulin secretion only during hyperglycaemia. When incretin-based therapies are used alone or with metformin, they possess lower risk of hypoglycaemia. In spite of inhibiting glucagon secretion during hyperglycemia, Vildagliptin is not known to compromise glucagon counterregulatory response during hypoglycemia in T1DM (Farngren et al., 2012). This is an advantageous characteristic of incretin mimetic and DPP-4 inhibitors, in contradiction to sulphonylureas, which promote insulin secretion irrespective of ambient glucose concentrations. In monotherapy trials, the overall extent of hypoglycaemia was akin to placebo for both vildagliptin and sitagliptin (Aschner et al., 2006). Sitagliptin also showed similar effects in combination with metformin and TZD. An exhaustive meta-analysis concluded that the sitagliptin is safe and was found to tolerable up to 2 years in clinical trials (Herman et al., 2010). Saxagliptin was responsible for reduced rates of hypoglycaemia, specifically when used in mono-therapy or in combination with metformin or TZD (Rosenstock et al., 2009b). In a study with vildagliptin supplemented insulin therapy, hypoglycaemic events were rare and less intense as compared to those receiving placebo plus insulin therapy (Fonseca et al., 2007). In another study, sitagliptin (100 mg once daily) reduced the risk of hypoglycaemia by six fold as compared to glipizide (Grunberger, 2014).

### 5.2 Weight gain

Contrary to the results acquired with GLP-1 mimics, no remarkable decrease in body weight has been observed with DPP-4 inhibitors either in monotherapy or combination therapy. These inhibitors seem to be body weight neutral (Ahren et al., 2004). DPP-4 inhibitors are associated with a very low risk of hypoglycaemia (DeFronzo et al., 2008b) and are not linked with weight gain, gastrointestinal symptoms or peripheral oedema. In mono-therapy or in combination with metformin, saxagliptin is weight neutral (Jadzinsky et al., 2009)^,^ (DeFronzo et al., 2009). And a combination of saxagliptin with glyburide display small but notable weight gain as compared with glyburide alone, along with improved glycaemic control (Chacra et al., 2009). No weight gain and decreased HbA1c levels were noted with vildagliptin also (Göke et al., 2008).

### 5.3 Effects on the immune system

In preclinical studies, it is evident that DPP-4 inhibitors can reduce inflammation (Shah et al., 2011a). Saxagliptin may also cause lymphopenia (Dhillon, 2015). There was minute reduction in the lymphocyte number induced by saxagliptin, yet mice medicated with saxagliptin and vildagliptin had no anomaly in their innate immune response fostered by Toll-like receptor ligands; cytokine creation, immune cell activation and lymphocyte trafficking were normal (Anz et al., 2014). Linagliptin treatment reduced inflammation and elevated epithelialization of wounds of diabetic mice (Schürmann et al., 2012). In human studies, evidence exists that sitagliptin possesses anti-inflammatory properties (Satoh-Asahara et al., 2013).

### 5.4 Cardiovascular effects

In terms of cardiovascular results, DPP-4 inhibitors are comparable to placebo (Fei et al., 2019). The previous studies did indicate cardiovascular benefits for DPP-4 inhibitors and a probable cause for this discrepancy is differences in characteristics of patients enrolled probably for the CV safety trials (Mannucci et al., 2016).

One such previous study indicated possible cardiac benefits (possibly mediated through GLP-1) and showed that DPP-4 inhibitors may also have direct effects on the heart and on cardiovascular risk factors (such as hypertension and hyperlipidaemia), independent of the incretin system (Zhang et al., 2019). The mechanism proposed was improved signalling via bone marrow chemokine which is known as SDF-1α, also named as pre-B cell growth stimulating factor (PBSF). Endothelial progenitor cells (EPCs), which are obtained from the bone marrow, are known to stimulate vascular repair and neo-angiogenesis. When vascular damage occurs, local growth factors and cytokines are convoluted which eventually signal the bone marrow to discharge EPCs targeted to the injured sites. EPCs develop into mature endothelial cells and support in vasculature reconstruction (Fadini and Avogaro, 2011). SDF-1α is one of the chief managers of EPCs stimulating their mobilisation (Heissig et al., 2002). Because SDF-1α is a well-known substrate for DPP-4, DPP-4 inhibition will increase the concentration of SDF-1α, potentially intensifying the transport of EPCs to injured endovascular sites (Packer, 2018). Encouraged by this concept, another likely mechanism is through a reduction in inflammation which is recognised as a main contributor to the atherosclerotic process. A few animal studies have also indicated that DPP-4 inhibition may lessen inflammatory cells within visceral adipose tissue and halt the progression of atherosclerosis via immunomodulation (Shirakawa et al., 2011).

In another study, DPP-4 treatment of both diabetic and non-diabetic hypertensive patients demonstrated lowering of blood pressure (Mistry et al., 2008). Sitagliptin helps the myocardium function after an ischemic episode and also improves overall cardiac performance in patients with coronary artery disease in the course of dobutamine stress echocardiography (Read et al., 2010). There is no cardiovascular risk with saxagliptin. On the contrary, it has potential for reduction in cardiovascular events (Frederich et al., 2010). Yet, these indications are preliminary, as the trials were not formulated to assess cardiovascular safety initially, even though the data indicates otherwise.

In total 20 clinical trials were analysed to reveal no enhanced risk of adverse cardiovascular effects or of heart failure in saxagliptin-treated patients (Iqbal et al., 2014). A study claims that T2DM patients with previous heart failure instances who were administered sitagliptin were more likely to be hospitalized for subsequent heart failure (Weir et al., 2014). Shah *et al.* revealed that DPP-4 inhibition within microcirculation mitigates vascular tone by direct mediation of the nitric oxide system (Shah et al., 2011b). The investigators proposed this drug promotes better control of blood pressure, which is a crucial cardiovascular risk factor. Also, DPP-4 inhibitors have not been found to have any noticeable effects on circulating lipid concentrations in human clinical trials. However, Boschman *et al.* (Boschmann et al., 2009) and Matikainen *et al.* (Matikainen et al., 2006) have reported the effects of vildagliptin therapy on post-prandial lipid mobilization, oxidation and lipoprotein metabolism in patients with T2DM, where DPP-4 inhibitor course ameliorated triglyceride and apolipoprotein B-48 particle digestion after a fat-rich meal. Thus it is clear that more studies need to elucidate the overall effects of DPP-4 inhibition on cardiovascular risk factors as the findings are limited and should be regarded as highly preliminary. In 2012 at Scientific Sessions of the American Diabetes Association (ADA), Johansen *et al.* presented the link between linagliptin and cardiovascular outcomes (Johansen et al., 2012).

### 5.5 Concern for pancreatitis and pancreatic cancer

A latest meta-analysis supervised by Monami *et al.* did not record any elevated risk of pancreatitis associated with DPP-4 inhibitors (Monami et al., 2014). No relationship between DPP-4 inhibitors and GLP-1RAs and pancreatitis and pancreatic cancer incidence has been established. This is supported by evidence from U.S. FDA and the European Medicines Agency (Egan et al., 2014).

### 5.6 Skin toxicity

After the approval of sitagliptin, serious allergic and hypersensitivity reactions were reported such as anaphylaxis, angioedema and exfoliated skin conditions like the Stevens–Johnson syndrome. In comparison to placebo, alogliptin-treated patients were more likely to report dermal side effects including pruritus (Rendell et al., 2012).

### 5.7 Outcomes in patients suffering from liver disease

An elevated DPP-4 mRNA expression in the livers of patients suffering from non-alcoholic fatty liver disease (NAFLD) is observed. Vildagliptin alone has shown hepatotoxicity in clinical trials. So far, hepatic impairment has not influenced vildagliptin pharmacokinetics (He et al., 2007b). To counteract this, it is advised that evaluation of liver enzymes be carried out within 3 months of initial vildagliptin treatment, and its usability is contraindicated in patients with serious liver disease. Dosage adjustment of linagliptin, alogliptin, saxagliptin, or sitagliptin is not recommended in patients with liver disease (Tella and Rendell, 2015).

### 5.8 Renal outcomes

Again, parallel to the cardiovascular effects, among the latest antidiabetic drug classes SGLT-2 inhibitors, GLP-1 RAs and DPP-4 inhibitors, SGLT-2 inhibitors are superior in terms of renal outcomes followed by GLP-1 RAs. DPP-4 inhibitors donot exhibit any beneficial renal outcomes (Fei et al., 2019). However, previous studies indicated no clear effect on kidney health (O’Hara et al., 2021). Except linagliptin, they are all eliminated renally and have been ascertained to accumulate in individuals with renal insufficiency. Sitagliptin is eliminated through urine via active tubular secretion and glomerular filtration in humans (Lyseng-williamson, 2007)^,^ (Scheen, 2010b). Patients with slight renal insufficiency did not demonstrate any clinically notable increase in the plasma concentration of sitagliptin. Sitagliptin was also moderately removed by haemodialysis. The US Food and Drug Administration (FDA) suggests the reduction of sitagliptin dose in patients with renal impairment, whereas the European Medicines Agency (EMA) advices to avoid sitagliptin in patients with moderate-to-severe or end-stage renal failure (Fasanya, 2021). Although there are no reports on the effect of saxagliptin on the renal system, the FDA suggests that renal function should be checked prior to and regularly after initiating saxagliptin therapy (Dhillon, 2015)^,^ (Food and Drug Administration, 2009). The pharmacokinetic properties of five DPP-4 inhibitors have been investigated in subjects with varying degrees of renal impairment (RI). Depending on the creatinine clearance they were classed as mild (50–80 mL/min), moderate (30–50 mL/min) and severe patients with end-stage renal disease (<30 mL/min) (Scheen, 2012a)^,^ (Scheen, 2010b).

• sitagliptin (25 mg and 50 mg—both moderate and severe renal impairment) (JANUVIA, 2020),
• saxagliptin (2.5 mg - both moderate and severe renal impairment) (OPDIVO, 2021),
• alogliptin (6.25 mg - severe renal disease including dialysis patients) (NESINA, 2013) and
• vildagliptin (50 mg - both moderate and severe renal impairment) (Novartis, 2012).

Linagliptin possesses a non-renal route of excretion and so can be employed without dose adjustment at all stages in patients with renal disease.

### 5.9 Other side effects

The most common side effects reported with sitagliptin include runny nose and sore throat, headache, diarrhoea, upper respiratory infection, joint pain and urinary tract infection. Hypersensitivity reactions such as Stevens-Johnson syndrome were also reported with sitagliptin (Fasanya, 2021). The following is a list of other adverse effects observed when sitagliptin was used in combination with.

• Metformin and rosiglitazone - headache, diarrhoea, and vomiting;
• Metformin - nausea;
• Pioglitazone - flatulence;
• Sulphonylurea and metformin - constipation; and
• Rosiglitazone or pioglitazone - peripheral edema.

When vildagliptin is combined with an angiotensin-converting enzyme inhibitor, some cases of angioedema have also been reported (Fasanya, 2021). The most common side effects seen were cold/flu-like symptoms, headaches and dizziness.

### 5.10 Gliptins in special populations

DPP-4 inhibitors are considered good options for older patients because of their efficacy and low risk of hypoglycaemia. Although, findings of these new agents are still scarce in this population area due to lack of proper representation in start of clinical trials, underlining the need for additional targeted studies (Scheen, 2012a). For elderly patients suffering from T2DM, lowering of HbA1c levels after treatment with DPP-4 inhibitors were not notably different from those observed in younger patients (Baron et al., 2006).

## 6 Implementing DPP-4 inhibitors in clinical practice

Utilising DPP-4 inhibitors in clinical practice is comparatively easy than other OADs as there is no need of dose titration and adjustment when used with other prescribed medications. In monotherapy, they could challenge traditional OADs. Saxagliptin is associated with appreciable drug–drug interactions, whereas both sitagliptin and vildagliptin demonstrate minor inclination for drug–drug interactions. As DPP-4 inhibition mainly supports physiological roles of endogenous GLP-1, these inhibitors may be of significant relevance in curing T2DM or even pre-diabetes, but this remains to be confirmed in comprehensive clinical trials. A review of Cochrane mentions the evaluation of sitagliptin and vildagliptin over a period of 12 and 52 weeks. The report disclosed lowering of HbA1c levels approximately 0.7% and 0.6%, respectively, in contrast to placebo (Richter et al., 2008b). Monami *et al.* carried out a meta-analysis comparing the surplus glycaemic effects of DPP-4 inhibitors and numerous anti-hyperglycaemic agents on the lipid profile in T2DM patients. It was observed that DPP-4 inhibitors were slightly more effective in lowering total cholesterol and triglycerides *vis-à-vis* other agents (Monami et al., 2012). DPP-4 inhibitors have a unique action pathway *vis-à-vis* other existing class of glucose lowering agents taken orally (Krentz et al., 2008). They enhance β-cell proliferation and neogenesis and also inhibit apoptosis (Butler et al., 2003). Apart from mono-therapy, DPP-4 inhibitors have shown promise in several combinations with other glucose-lowering agents (Neumiller et al., 2010). Combination of DPP-4 inhibitors with existing drugs could be a welcome supplement. The most favoured combination of DPP-4 inhibitors is with metformin (Dahut and Madan, 2010). Combining metformin/DPP-4 inhibitor resulted in higher GLP-1 concentrations compared to DPP-4 inhibitor mono-therapy (Vardarli et al., 2014). Rather in one of the study, metformin itself acts as a DPP-4 inhibitor by inhibiting the DPP-4 enzyme in the fasting state (Cuthbertson et al., 2009). Moreover, with this dual therapy (metformin/DPP-4 inhibitor), patients can also receive additional treatment intensification with sodium-glucose cotransporter-2 (SGLT-2) inhibitor or thiazolidinedione (TZD) (Inzucchi et al., 2015). Other combinations include sulphonylurea, thiazolidinedione or metformin and sulphonylurea combined therapy (Drucker and Nauck, 2006).

Combination of GLP-1RAs with DPP-4 inhibitors is not advisable, because no clinically significant auxiliary benefit is predicted, although there are no concerns about adverse effects (Davies et al., 2018); (American Diabetes Association, 2019). Patients unable to tolerate GLP-1RAs treatment are recommended for DPP-4 inhibitor therapy. Also, it is advised that DPP-4 inhibitor therapy should cease when treatment is taken forward with an injectable (GLP-1RAs) therapy, at later stages (American Diabetes Association, 2019).

### 6.1 Monotherapy

Metformin has solidified its place as the first-line drug for treating T2DM (Rodbard et al., 2009). Predominantly, metformin exhibited slight but notable reductions in both HbA1c and body weight. On the other hand, the DPP-4 inhibitor demonstrated superior gastrointestinal tolerability as compared to metformin. Overall, the reduction in HbA1c was same with both the pharmacological approaches, with low likelihood of hypoglycaemic events (Scheen, 2012c).

#### 6.1.1 Vildagliptin monotherapy

A 12-week investigation was designed to ascertain a dosage of vildagliptin which was effective in lowering down HbA1c levels and was safe and well tolerated in type 2 diabetic patients (baseline HbA1c 7.6%–7.8% and fasting plasma glucose 9.2–9.4 mmol/L for vildagliptin vs placebo, respectively). There was statistically notable reduction in HbA1c levels in the vildagliptin 50 and 100 mg/day groups as compared to placebo (Ristic et al., 2005).

#### 6.1.2 Sitagliptin mono-therapy

The clinical inquiry of sitagliptin followed a similar design to the vildagliptin study. In two 12-week dose-range studies, all sitagliptin doses markedly reduced HbA1c compared with baseline. In another study of diabetic patients who had also undergone coronary heart disease, it was demonstrated that treatment with sitagliptin improved their heart function and coronary artery perfusion, validated by echo-debutamin tests (Read et al., 2010). For coronary heart disease threat factors, DPP-4 may rise in response to fall in blood pressure. Mistry *et al.* displayed that sitagliptin produced little but statistically notable reductions in blood pressure (Mistry et al., 2008). Marney et al. hypothesised that the combination of sitagliptin with high-dose angiotensin-converting enzyme (ACE) inhibition leads to activation of sympathetic tone and thus attenuates blood pressure reduction process (Marney et al., 2010).

#### 6.1.3 Saxagliptin mono-therapy

Saxagliptin showed significant reduction in HbA1c levels for 2.5, 5 and 10 mg vs placebo in a main treatment cohort of 401 patients over a period of 24 weeks. Likelihood of adverse reactions was similar in both placebo and saxagliptin treated patients. No weight gain or hypoglycaemia cases were observed (Rosenstock et al., 2009b). Another study focusing cardiovascular effects concluded that although no increased risk of ischemic stroke was related to saxagliptin use, hospitalisation rate due to heart failure did increase. The study recommended other approaches to minimise this cardiovascular risk (Scirica et al., 2013).

### 6.2 Gliptins in special combinations

The combination treatment of DPP-4 inhibitors with some other anti-hyperglycaemic drugs may be worthwhile as these agents target contrasting pathophysiological processes and would be anticipated to have additional benefits on measures of glycaemic control (Barnett, 2006). Because of cost, lack of familiarity and no endpoint data, they ought to be employed mainly in combination treatment, in the initial years.

#### 6.2.1 Gliptins combined with sulphonylureas

While the combination of gliptin–metformin did not result in hypoglycaemia, such events may arise with the gliptin–sulphonylurea combination. Therefore, in patients with T2DM characterised with moderately elevated HbA1c levels taking sulphonylurea as monotherapy, it would be safer to reduce the sulphonylurea dose when a DPP-4 inhibitor is added on. This would reduce the risk of hypoglycaemia, especially in elderly patients (Scheen, 2012c).

#### 6.2.2 Gliptins combined with thiazolidinediones

The impact of combination of sitagliptin-metfromin vs pioglitazone monotherapy was studied in a controlled trial. The subjects were patients with poorly controlled T2DM. Improvements in HbA1c, fasting blood glucose and postprandial glucose levels were noted. Metformin caused body weight reduction, and swift improvement of insulin resistance and inflammation parameters. In contrast, sitagliptin performed better on the conservation of β-cell function. This research suffered a drawback since the dose of pioglitazone employed was non-identical between the two trial arms, i.e. 15 mg with metformin and 30 mg with sitagliptin (Derosa et al., 2010a). In another study, pioglitazone was taken in combination with vildagliptin and it was even more effective in preserving β-cell function and reducing insulin resistance and inflammation parameters. Similar improvements in glucose control parameters as compared to glimepiride-vildagliptin combination was observed (Derosa et al., 2010b). DPP-4 inhibitor with pioglitazone combination thus is a promising avenue for patients who cannot tolerate either metformin or sulphonylurea as an effective and safe therapeutic approach (Mikhail, 2008).

#### 6.2.3 Gliptins as oral triple therapy

Initial intervention with a combination therapy of vildagliptin plus metformin delivers better and robust longstanding benefits in comparison to the present standard-of-care initial metformin monotherapy for patients with newly diagnosed T2DM as proved by the VERIFY study (Matthews et al., 2019). The advent of DPP-4 inhibitors offered new candidates for oral triple therapy at a time when the metformin-sulphonylurea-TZD combination was the only available option (Scheen et al., 2009).

• Sitagliptin daily dosage remarkably improved glycaemic control and β-cell function in T2DM patients who had ineffective glycaemic control with glimepiride and metformin therapy (Hermansen et al., 2007).
• Combining linagliptin with metformin and with sulphonylurea separately, significatly improved glycaemic control in patients with T2DM and was well tolerated (Owens et al., 2011).
• Combining alogliptin with metformin–pioglitazone combination stimulated better glycaemic control and potentially improved β-cell function (Bosi et al., 2011).

#### 6.2.4 Gliptins combined with insulin in type 2 diabetes mellitus

DPP-4 inhibitors have advantages such as lack of hypoglycaemia and weight gain which justifies combining DPP-4 inhibitors with insulin. In addition to that, a DPP-4 inhibitor decreases glycaemic excursions that are not appropriately addressed by basal insulin therapy even after optimization of the titration of the basal insulin. Combining a DPP-4 inhibitor with insulin therapy may be useful in patients with T2DM for improving glucose control without prompting hypoglycaemia and likely suitable for restricting weight gain. Further studies are desirable to explore the role of DPP-4 inhibitor added to optimized insulin scheme, as the available research only involved patients using basal insulin therapy (Charbonnel et al., 2013).

#### 6.2.5 Gliptins in initial combination therapy

Gliptins as initial combinations may offer a complementary initial treatment option for T2DM, especially in cases where assigning metformin is precarious such as in patients with renal impairment.

### 6.3 Head-to-head trials comparing incretin-based therapies

#### 6.3.1 DPP-4 inhibitors vs GLP-1 receptor agonists

GLP-1 RAs mimic endogenous GLP-1, while DPP-4 inhibitors block their degradation (Neumiller, 2009). Thus both these approaches elevate GLP-1 activity and improve plasma glucose control. Though, head-to-head comparisons of the two therapies are scant and only one DPP-4 inhibitor, i.e., sitagliptin has been so evaluated, in meta-analyses of randomized controlled trials, GLP-1 receptor agonists were better in reducing blood glucose and resulted in substantial weight loss, whereas DPP-4 inhibitors reduces blood glucose levels to a lesser extent and are weight-neutral (Fakhoury et al., 2010). GLP-1 receptor agonists showed superiority over DPP-4 inhibitors, yet the average moderate differences in HbA1c and weight reductions may be counteracted by numerous disadvantages of GLP-1RAs (Gilbert and Pratley, 2020). However, generally GLP-1 RA’s are preferred over DPP-4 inhibitors.

## 7 DPP-4 inhibitors - Future prospects

New incretin-based therapies provide numerous options for glycaemic control in patients with T2DM and have clear advantages over other classical glucose-lowering agents (Phung et al., 2010), yet estimated cost of the therapy is an essential factor when making global comparisons for clinical usage (Waugh et al., 2010). DPP-4 inhibitors undoubtedly are more expensive than sulphonylureas, but more affordable than GLP-1 receptor agonists. At the moment, only sparse preliminary data are available (Schwarz et al., 2008). Thus, further economic analyses are necessary to support the switch from sulphonylureas to DPP-4 inhibitors (Waugh et al., 2010). In addition to this, further studies are needed to explore the long-term safety and efficacy of DPP-4 inhibitors. This will help establish their role in the management of T2DM and provide clinicians with valuable information to make informed treatment decisions. Additionally, identifying patient populations that are most likely to benefit from DPP-4 inhibitor therapy is crucial for personalized treatment plans. This can be achieved through ongoing clinical trials and real-world evidence studies. Furthermore, research is needed to investigate the potential benefits of combination therapy with DPP-4 inhibitors and other glucose-lowering agents. This could help improve overall glycemic control and reduce the risk of adverse effects associated with higher doses of single agents. Overall, ongoing research in the field of DPP-4 inhibitors holds promise for improved management of T2DM and better patient outcomes.

## 8 Conclusion

Leveraging incretin effect for treating T2DM is a relatively novel line of treatment. DPP-4 inhibitors are rapidly catching attention as they not only complement but also extend the traditional therapeutic avenues to treat T2DM. Improvement in T2DM in terms of delay in gastric emptying, reduction in postprandial glucose levels, and inhibition of glucagon secretion is observed with DPP-4 inhibitors. As recounted above, they can be efficiently used in combination therapy. Moreover, they have the advantage of meeting medical needs in metabolic disorders, which is especially significant for elderly diabetic patients or patients having obesity problems along with T2DM. In addition to that, evidence increasingly points towards reversal of dysfunction of pancreatic *β* cells. It is believed that the anti-apoptotic and proliferative characteristics of DPP-4 inhibitors can help in the regeneration of pancreatic β cells. Research also needs to be focused on finding the correct intervention point to mitigate progressive loss of *β*cells mass and function—the hallmark of prediabetes.

Though many questions have been raised regarding the safety profile of DPP-4 inhibitors particularly in areas of pancreatitis and pancreatic cancer, not much evidence is available for it. One should be cautious nonetheless and further research is needed in this regard. GLP-1 stimulation, at this stage should prove beneficial but again little evidence exists to support this hypothesis and more studies are needed for clarification in this regard.

The most disappointing result is its negligible favourable effect on cardiovascular and renal outcomes compared to other new antidiabetic agents like SGLT-2 inhibitors or GLP-1 RAs.

Apart from the cyanopyrollidine scaffolds mentioned above, research groups and pharmaceutical companies all over the world are developing multiple new candidates, many of which are already undergoing clinical trials. This is testament to the importance of harnessing the incretin effect as an attractive approach for T2DM therapy, as well as the role of DPP-4 as a drug target in achieving this goal.

The best treatment option for diabetes would not only control blood sugar levels but would also help in managing overall risk factors. In this context, DPP4 inhibitors seem to be very good candidates as are not known to cause hypoglycemia or weight gain, no requirement of dose escalation and have a favorable anti-inflammatory and safety profiles. Also, they are relatively safer for elderly diabetic patients and kidney disease patients with diabetes. They have been used quite extensively and thus there is lot of experience in their use. All these factors make them excellent choices and it can be safely said that all the hype surrounding DPP-4 inhibitors is very well justified.
